# Primary palliative care recommendations for critical care clinicians

**DOI:** 10.1186/s40560-022-00612-9

**Published:** 2022-04-15

**Authors:** Kaori Ito, Naomi George, Jennifer Wilson, Jason Bowman, Emily Aaronson, Kei Ouchi

**Affiliations:** 1 Department of Emergency Medicine, Division of Acute Care Surgery, Teikyo University School of Medicine, Tokyo, Japan; 2 Department of Emergency Medicine, University of New Mexico, Albuquerque, NM USA; 3 Department of Emergency Medicine, Stanford University School of Medicine, Stanford, CA USA; 4 Department of Emergency Medicine, Brigham and Women’s Hospital, 75 Francis St, Neville 200, Boston, MA 02125 USA; 5 Department of Emergency Medicine, Harvard Medical School, Boston, MA USA; 6 Department of Psychosocial Oncology and Palliative Care, Dana-Farber Cancer Institute, Boston, MA USA; 7 Department of Emergency Medicine, Massachusetts General Hospital, Boston, MA USA; 8 Serious Illness Care Program, Ariadne Labs, Boston, MA USA

## Abstract

Palliative care is an interdisciplinary care to optimize physical, psychosocial, and spiritual symptoms of patients and their families whose quality of life is impaired by serious, life-limiting illness. In 2021, the importance of providing palliative care in the intensive care unit (ICU) is well recognized by various studies to alleviate physical symptoms due to invasive treatments, to set patient-centered goals of care, and to provide end-of-life care. This paper summarizes the evidence known to date on primary palliative care delivered in the ICU settings. We will then discuss the potential benefits and harms of primary palliative care so that critical care clinicians are better equipped to decide what services might best improve the palliative care needs in their ICUs.

## Introduction

Palliative care is an interdisciplinary care to optimize physical, psychosocial, and spiritual symptoms of patients and their families whose quality of life is impaired by serious, life-limiting illness. Up to 75% of patients admitted to the intensive care units (ICU) experience distressful symptoms [1] (e.g., 57% with traumatic stress, 80% with anxiety and depression, etc. [2]); thus, 20% of such patients may require palliative care consultations [3]. In the U.S., inventions of life support technology in the 1960s (e.g., ventilators) led to widespread development of ICUs nationwide. Yet in the 1970s, it became apparent that many patients were dying in ICUs after life-prolonging treatment. In the 1980s and 1990s, critical care clinicians began to provide palliative care interventions for “hopelessly ill” patients [4]. In 2021, the importance of providing palliative care in the ICU is well recognized by various studies to alleviate physical symptoms due to invasive treatments, to set patient-centered goals of care, and to provide end-of-life care [5].

Two main provisions of palliative care exist: primary palliative care and specialty palliative care. Primary palliative care is provided by clinicians who are not subspecialty-trained in palliative care and provide front-line care to patients. On the other hand, specialty palliative care is provided by qualified, subspecialty-trained, palliative care clinicians [6, 7]. Given the shortage of these subspecialty-trained, palliative care clinicians, the recent focus is on increasing the implementation of primary palliative care interventions in the critical care settings. Although there have been several studies on primary palliative care by critical care clinicians, the results have been controversial, and it is not clear what primary palliative care interventions are most appropriate in the ICU settings.

Recently, in the U.S., there has been a growing body of knowledge regarding palliative care for acutely ill patients in emergency and ICU settings [8]. This paper summarizes the evidence known to date on primary palliative care delivered in the ICU settings. We will then discuss the potential benefits and harms of primary palliative care so that critical care clinicians are better equipped to decide what services might best improve the palliative care needs in their ICUs (Table 1).

**Table 1:** Recommended system of the primary palliative care in the intensive care unit

Target	Interventions	References
Patient	Basic symptom relief for fatigue, thirst, and pain Prevention for the post-ICU syndrome The timing of initiation of the palliative care should be tailored based on the trajectory of the illness	[1, 9–12] [13–20] [21]
Family	Patient-/family-centered decision-making Emotional and practical support Structured family communication and brochures for families The introduction of a communication facilitator or family support coordinator to support the primary team and facilitate structured communication	[23] [23] [24–27] [25]
Clinician	Education about palliative care (didactic and simulation trainings) Bedside tools and techniques Real-time collaboration and feedback with subspecialty-trained palliative clinicians Communication skills training for the goals-of-care discussion Implement multifaceted bundles to improve critical care clinicians’ ability to provide palliative care Palliative care interventions on critical care clinician wellness “Death rounds” in the ICU	[39, 40] [39, 40] [39, 40] [41–47] [48, 49] [50] [51, 52]
System-level	Triggered palliative consultations Simulations for intensivists to record their estimate of a patient’s 3-month functional outcome The implementation of an order set focused on the care processes surrounding withdrawal of life-sustaining treatment (including preparations, sedation/analgesia, withdrawal of ventilation and principals of life support)	[57, 58] [59] [60, 61]
Multilevel	Family-facing: scheduled, end-of-life conferences and bereavement brochure + Clinician-facing: communication skills training for goals-of-care conversations System-level: triggered palliative care consultations + Clinician-facing: palliative care assessment form in the medical records + Family-facing: family-involvement in decision-making with the use of time-limited trial System-level: hospital policy for a three-tiered classification for the intensity of care/resuscitation, comprehensive care team evaluation + Family-facing: family-involvement in decision-making Clinician-facing: a 12-h communication skills training for ICU nurse champion + Family-facing: daily, structured family support meetings + System-level: implementation specialist for each ICU to incorporate the above into regular workflow	[30] [62] [63] [64]

### Palliative care interventions for patients in the ICU

One of the primary missions of the ICU is to save the patient's life. Therefore, some critical care physicians may be reluctant to apply palliative care for critically ill patients, which may seem contradictory to ICU’s mission by forgoing intensive, life-saving treatment. In fact, such prejudice against palliative care may potentially hindered its implementation in ICUs. However, since the 1990s, patients in the ICU, especially those on ventilator management, have been experiencing various symptoms, and the need for palliative care in terms of physical, psychosocial, and social aspects has been drawing increasing attention.

Among ICU patients, 80% reported experiencing fatigue, 85% thirst, 60% pain, and 75% weight loss [1, 9–12]. Moreover, studies of ICU survivors have shown that physical symptoms resulting from serious illness can persist for months or even years after ICU discharge which are described as the post-ICU syndrome [13–20]. These findings underscore the importance of palliative care for patients' physical symptoms only in the ICU, but also after discharge from the ICU.

As mentioned above, ICU patients suffer from a variety of physical symptoms. Therefore, invasive treatments and palliative care should be provided simultaneously in the ICU. When palliative care in the ICU should be initiated is likely to vary depending on the illness trajectory [21]. For example, in the case of conditions that lead to sudden death, such as cardiovascular disorders and trauma, palliative care should probably be concurrent with intensive care, since these conditions are often the end of life within a few days of habitual ones. In the case of patients with organ failure (e.g., cirrhosis, chronic obstructive pulmonary disease, and chronic heart failure patients), function gradually declines with repeated remissions and exacerbations. They often have a rapid decline in function just prior to death, and in such cases, both intensive care and palliative care interventions will be necessary. For frail patients, it may be difficult to determine where they are at the end of life, as their function declines on a yearly basis. When such patients are admitted to the ICU with some serious illness, the indication for invasive treatment should be carefully judged based on the patient's values, and palliative care should be offered in conjunction with intensive medical care.

To avoid the perception of conflict of interest, physicians on the care team generally do not mention organ donation to the families in the U.S. This task is typically delegated to an unbiased, a third-party coordinator (e.g., regional or national organ donation centers) who introduces the concept of organ donation. Occasionally, a palliative care physician will continue this conversation with the family of a patient who has been declared brain dead about what happens next. Some data exist that communication training may be helpful for palliative care physicians to discuss this topic with the families of brain-dead patients [22].

### Palliative care interventions for patients’ families in the ICU

Many palliative care interventions in the ICU have focused on supporting families of ICU patients, specifically targeting the domains of patient-/family-centered decision-making, emotional and practical support, and communication [23]. Researchers have evaluated several different types of primary palliative care interventions, including structured family communication and brochures for families. Some studies have involved the introduction of a communication facilitator or family support coordinator to support the primary team and facilitate structured communication [24–27]. The most common study designs were pre- and post-intervention comparisons and randomized clinical trials. Outcomes of interest have included frequency and duration of communication with ICU providers, patient/family satisfaction, depression and anxiety scores, likelihood of choosing to forego resuscitation or pursue comfort care, ICU length of stay, and mortality [28].

Overall, effects of these primary palliative care interventions on family satisfaction and anxiety/depression scores have been mixed, with some studies showing modest improvements and others showing no difference between groups [24–26, 29–32]. The reason why family satisfaction may not improve with the intervention is that the family's admission to the ICU with a serious illness, and possibly death, is the worst thing that can happen to them. The family would have no counterfactual outcome to compare their experience to.

In contrast to the mixed results observed in family satisfaction and psychological distress, multiple studies have found that family-focused, primary palliative care interventions are consistently associated with decreased critical care use at the end of life [25, 27, 32–36]. ICU length of stay is reduced on the order of one to four days in most of these studies, driven by shorter duration of critical care among patients who died. This effect is likely mediated by an increase in families opting to forego resuscitation or pursue comfort care-only approach and doing so at an earlier stage [26, 27, 36]. Importantly, multiple studies have demonstrated that overall ICU and in-hospital mortality is not increased by these interventions [25, 27, 33, 37]. While White et al. did find higher in-hospital mortality among participants assigned to the family support intervention arm in their trial (38.0% vs 30.2% in the control arm), there was no difference in mortality at six months.

Taken together, these studies suggest that while family-focused primary palliative care interventions in the ICU have variable effects on family satisfaction and psychological distress, they are a cost-effective intervention for reducing potentially undesired critical care at the end of life and do not seem to increase overall mortality. Future primary palliative care research should investigate why some family support interventions seemed to improve family satisfaction and psychological distress, and to elucidate which interventions accomplish both improving the family experience and reducing intensity of care at the end of life. As discussed below, it may be that complementary clinician-focused or systems-based interventions improve these outcomes as well.

### Palliative care interventions for clinicians in the ICU

The impact of interventions on clinicians has also been increasingly explored and studied over the past several decades [38]. These interventions include didactic and simulation trainings, bedside tools and techniques, and real-time collaboration and feedback with subspecialty-trained palliative clinicians. As with other interventions described in this chapter, outcomes have been mixed, likely in part a result of heterogeneous populations of clinicians, patients, and families, as well as the difficulty in measuring some important but nuanced outcomes.

In a 2014 national study of 89 hospitals and 71 ICU training programs, quality of education in primary palliative care skills and presence of evidence-based bedside tools were both associated with reduced ICU length of stay [39]. Several other studies have shown a similar effect on length of stay, as well as that education increases the rate of palliative care consultation. Educational interventions have also been shown to increase critical care clinicians’ comfort with palliative care topics and also the likelihood that they will engage in discussions about them with patients and families [40].

Recently, the importance of communication skills training for critical care clinicians has been attracting attention. Oppenheim et al. reported that in a simulated conversation between an ICU health care provider and a patient's family regarding the patient's prognosis, family members interpret physicians’ indirect response to questions about prognosis as more optimistic than direct responses [41]. Therefore, it is important for critical care clinicians to clearly communicate the prognosis in conversations with the patient's family and then set goals of care that are aligning with the patient's values, including the initiation of palliative care.

Critical care clinicians receive family-centered communication skills training as part of their ICU training, which can improve healthcare professionals' self-efficacy and family satisfaction [42]. In the U.S., educational methods such as Vital Talk are known as communication skills training methods [43]. Vital Talk method was originally developed for communicating with cancer patients, but has been applied to a variety of fields and has even been developed for emergency physicians and critical care clinicians [44, 45]. The authors developed Vital Talk in Japanese language and found that it improved learners' preparedness to communicate with critically ill patients [46, 47].

Attempts to implement multifaceted bundles to improve critical care clinicians’ ability to provide palliative care have had mixed results. For example, the largest such project published to date showed a positive outcome in the initial single site study, but no difference in the subsequent multisite study [48, 49].

Finally, relatively little has been published about the impact of palliative care interventions on critical care clinician wellness. One single site study showed that trainees felt palliative care involvement was supportive for them and helped them be better clinicians [50]. “Death Rounds”, collaboratively reviewing the cases of patients who have died and the clinicians’ experiences of caring for them, are common within palliative care programs. More recently, this tool has also been implemented in ICUs, with attendees reporting feeling more supported, a sense of closure, better prepared for similar future cases, and that tools like this should be more universally incorporated in ICUs [51, 52].

### System-level, palliative care interventions in the ICU

In addition to interventions targeted individual critical care clinicians, there have been some interventions aimed at designing systems to help support the reliable delivery of palliative care in the ICU. The idea of system design as a lever to facilitate best-care, has been well documented in the ICU literature [53]. The interventions described have predominantly been aimed at systems that help mitigate against infection risk, or try to ‘nudge’ clinicians towards behaviors that make care more efficient [54–56]. More recently, however, there have been several small studies of system interventions that drive the adoption of palliative care principles. One studied intervention is related to triggered consults. In one pre/post-study of 81 patients with dementia, a triggered palliative care consult was demonstrated to decrease ICU and hospital length of stay, and to result in less interventions for DNR patients [57]. In a larger prospective, multicenter RCT of triggered ethics consults, those randomized to the triggered consult had a reduced ICU and hospital lengths of stay and less mechanical ventilation days [58]. Triggered palliative care consultations are different from the traditional case-by-case consultations in that they are automatically triggered when eligible patients exist (e.g., dementia patients). The triggered consultation practice has led to more consultations. As previously demonstrated in other settings, the increase in palliative care consultations may have reduced the use of ICU resources by finding more patients who preferred to stay away from invasive treatment. In addition to triggered consults, other system-level interventions have been explored. In one small, simulation-based study, it was found that by creating a system in which intensivists had to record their estimate of a patient’s 3-month functional outcome they were significantly more likely to discuss the withdrawal of life-sustaining treatment in a hypothetical family meeting [59]. Lastly, technology-enabled interventions have also been investigated, with two quality improvement studies describing the implementation and looking at the impact of order sets. The first study looked at the implementation of an order set focused on the care processes surrounding withdrawal of life-sustaining treatment (including preparations, sedation/analgesia, withdrawal of ventilation and principals of life support) on clinician satisfaction, the use of analgesia, and nurses’ perception of the patients’ dying experience. Although clinicians found the form helpful and the use of opiates and benzodiazepines increased, it did not change nurse assessment of the patient’s dying experience [60]. Interestingly, in a similarly small quality improvement study looking at the impact of a withdrawal of life support protocol, the number of comfort medications decreased, and the pastoral care involvement increased [61]. This study also aimed to assess the impact on the time between ICU admission to withdrawal of life support, which decreased with the implementation of the new protocol.

In summary, although several exploratory or quality improvement studies have investigated the impact of system interventions to drive best-care, and some suggest promise, none have demonstrated a system solution that yet warrants wide-scale adoption.

### Multi-level, palliative care interventions in the ICU

Multi-level ICU palliative care interventions are interventions that include components from across several domains (i.e., family-oriented, clinician-facing, and system-level interventions) and frequently target multiple outcomes. As detailed above, family-, clinician-, and system-level interventions individually have yielded some promising results to improve the overall quality of care. However, the impact on patient/surrogate-centered outcomes has been limited in many trials. The motivation for multi-level interventions is to leverage the benefits found in single-level interventions and to extend the scope or reach of those benefits by addressing theoretical barriers to improvement. Multi-level ICU interventions are variably designed, with outcomes often set to test a combination of surrogates’ psychological distress, ICU utilization metrics, and quality of communication measures. At their core, most focus on addressing communication and information exchange [30, 62].

Nearly all multi-level palliative care ICU trials have shown significant reductions in ICU length of stay without increasing mortality [30, 62, 63]. However, even here some exceptions existed, demonstrating that some well-designed trials may not reduce ICU utilization [49]. Some of the most impressive results come from a multicenter trial using a combination family brochure and proactive family meeting strategy, which showed a significant reduction in surrogate psychological distress during bereavement [30]. However, this study did not address surrogates of ICU survivors, and generalizability outside the French context may be limited. Another key multi-level intervention study was the PARTNER trial [64], a step-wedge, cluster randomized trial comparing a multi-component family support intervention to usual care to reduce long-term psychological burden of surrogates of critically ill patients. In this trial, 1420 patients were randomized to either the intervention: a multistep patient/surrogate support pathway implemented by ICU nurses or usual care. While the authors found no difference in the primary outcome (surrogate anxiety and depression at 6 months) they noted improvement in several important secondary outcome measures including higher ratings of ICU communication quality by surrogates, and higher ratings on the Patient Perception of Patient Centeredness scale. Finally, it is important to recognize that at least one multi-level palliative care interventions did show a slight signal of harm [37]. In this study, a combination ICU brochure and palliative care-led information and support meetings intervention was associated with higher levels of post-traumatic stress disorder in the intervention group. The intervention included a focus on patient prognosis during family meetings. The authors conclude that this component of the intervention could have been distressing to surrogates and led to higher rates of post-traumatic stress disorder.

Taken together, multi-level trials hold promising results, particularly in improving surrogate psychological symptoms as compared to single-level interventions. However, even here the scope and impact of palliative ICU interventions could be improved. Several theoretical limitations may explain the variable or limited impact of multi-level interventions. First, few interventions have included components that target the post-ICU period, when bereavement and other challenges may pose a substantial burden for surrogates [65]. Second, it may be that financial hardship, caregiving burden, and other psychosocial factors are more important drivers of surrogate psychological outcomes than ICU communication [66]. Thus, the ability for a multi-level ICU intervention to improve psychological outcomes is inherently limited. Finally, it will be important to demonstrate through implementation and dissemination studies, that these multi-system interventions can be replicated outside of the research setting. Incorporation of system-level interventions, for instance electronic health record-triggered palliative care consultations, may be particularly important to implementation.

### Risks and benefits of primary palliative care implementations in the ICU

We have described the potential benefits of primary palliative care interventions that focus on the families, clinicians, and system levels. Many demonstrated modest effects on the overall quality of care yet often lacked patient-centered benefits. What about the potential harms of implementing these interventions in the ICU settings? The main costs of implementations are clinician training, clinical time to deliver primary palliative care services, and system-level tools (e.g., electronic health record triggers for palliative care needs). The potential barriers [67–69] and risks [37, 70, 71] of the implementation of the palliative care in the ICU are summarized in Table 2. In comparison to other healthcare interventions (e.g., clinical pharmacist) [72], training of critical care clinicians and delivery of primary palliative care seem insubstantial, especially in the face of cost-saving that hospitals can expect. Thus, the impetus for implementing primary palliative care hinges on the healthcare organizations’ willingness to invest in relatively low-cost palliative care interventions for likely overall benefit in the quality of care and healthcare utilization costs.

**Table 2:** Barriers and risks of the implementation of the palliative care in the intensive care unit

Barriers	References
Critical care clinicians are not aware of the palliative care needs of ICU patients due to competing demand Inadequate palliative care screening for ICU patients Difficulty in communicating adequately with the patient's family at the right time Clinician concerns regarding palliative care hastening death Inadequate palliative care training for ICU medical staff Palliative care staff unavailability Patient/family misconception of palliative care Time and cost to train critical care clinicians for the palliative care	[67–69]

Risks	References
Increasing post-traumatic stress disorder in the patient’s surrogates when discussing about the patient’s prognosis Given the baseline severity of illnesses, harmful consequences from medical errors in patients receiving palliative care may be harder to identify	[37] [70, 71]

## Conclusion

In summary, we described the modest benefits of primary palliative care interventions in the ICU settings. Overall improvement in quality of care can be expected despite the mixed level of evidence in patient-centered outcomes. Given the low potential harm and cost of implementing these primary palliative care interventions, healthcare organizations may consider initial investment to disseminate some forms of primary palliative care interventions in the ICU settings.

## Data Availability

Not applicable.

## References

[1] Puntillo KA, Arai S, Cohen NH, Gropper MA, Neuhaus J, Paul SM, Miaskowski C (2010). Symptoms experienced by intensive care unit patients at high risk of dying. Crit Care Med.

[2] McAdam JL, Fontaine DK, White DB, Dracup KA, Puntillo KA (2012). Psychological symptoms of family members of high-risk intensive care unit patients. Am J Crit Care.

[3] Hua MS, Li G, Blinderman CD, Wunsch H (2014). Estimates of the need for palliative care consultation across united states intensive care units using a trigger-based model. Am J Respir Crit Care Med.

[4] Miedema F (1993). Withdrawing treatment from the hopelessly ill. Part 1: The ethical case. Dimens Crit Care Nurs.

[5] Aslakson RA, Curtis JR, Nelson JE (2014). The changing role of palliative care in the ICU. Crit Care Med.

[6] Quill TE, Abernethy AP (2013). Generalist plus specialist palliative care-creating a more sustainable model. N Engl J Med.

[7] Weissman DE, Meier DE (2011). Identifying patients in need of a palliative care assessment in the hospital setting: a consensus report from the Center to Advance Palliative Care. J Palliat Med.

[8] George N, Bowman J, Aaronson E, Ouchi K (2020). Past, present, and future of palliative care in emergency medicine in the USA. Acute Med Surg..

[9] Puntillo KA (1990). Pain experiences of intensive care unit patients. Heart Lung.

[10] Puntillo K, Nelson JE, Weissman D, Curtis R, Weiss S, Frontera J, Gabriel M, Hays R, Lustbader D, Mosenthal A, Mulkerin C, Ray D, Bassett R, Boss R, Brasel K, Campbell M (2014). Palliative care in the ICU: relief of pain, dyspnea, and thirst—a report from the IPAL-ICU Advisory Board. Intensive Care Med.

[11] Li L, Nelson JE, Hanson LC, Cox CE, Carson SS, Chai EJ, Keller KL, Tulsky JA, Danis M (2018). How surrogate decision-makers for patients with chronic critical illness perceive and carry out their role. Crit Care Med.

[12] Nelson JE, Meier DE, Litke A, Natale DA, Siegel RE, Morrison RS (2004). The symptom burden of chronic critical illness. Crit Care Med.

[13] Choi J, Hoffman LA, Schulz R, Tate JA, Donahoe MP, Ren D, Given BA, Sherwood PR (2014). Self-reported physical symptoms in intensive care unit (ICU) survivors: pilot exploration over four months post-ICU discharge. J Pain Symptom Manage.

[14] Choi J, Tate JA, Hoffman LA, Schulz R, Ren D, Donahoe MP, Given BA, Sherwood PR (2014). Fatigue in family caregivers of adult intensive care unit survivors. J Pain Symptom Manage.

[15] Cox CE, Docherty SL, Brandon DH, Whaley C, Attix DK, Clay AS, Dore DV, Hough CL, White DB, Tulsky JA (2009). Surviving critical illness: acute respiratory distress syndrome as experienced by patients and their caregivers. Crit Care Med.

[16] Herridge MS, Tansey CM, Matte A, Tomlinson G, Diaz-Granados N, Cooper A, Guest CB, Mazer CD, Mehta S, Stewart TE, Kudlow P, Cook D, Slutsky AS, Cheung AM, Canadian Critical Care Trials G (2011). Functional disability 5 years after acute respiratory distress syndrome. N Engl J Med.

[17] Iwashyna TJ, Ely EW, Smith DM, Langa KM (2010). Long-term cognitive impairment and functional disability among survivors of severe sepsis. JAMA.

[18] Fan E, Dowdy DW, Colantuoni E, Mendez-Tellez PA, Sevransky JE, Shanholtz C, Himmelfarb CR, Desai SV, Ciesla N, Herridge MS, Pronovost PJ, Needham DM (2014). Physical complications in acute lung injury survivors: a two-year longitudinal prospective study. Crit Care Med.

[19] Dowdy DW, Eid MP, Dennison CR, Mendez-Tellez PA, Herridge MS, Guallar E, Pronovost PJ, Needham DM (2006). Quality of life after acute respiratory distress syndrome: a meta-analysis. Intensive Care Med.

[20] Boyle M, Murgo M, Adamson H, Gill J, Elliott D, Crawford M (2004). The effect of chronic pain on health related quality of life amongst intensive care survivors. Aust Crit Care.

[21] Lunney JR, Lynn J, Foley DJ, Lipson S, Guralnik JM (2003). Patterns of functional decline at the end of life. JAMA.

[22] Kramer NM, O'Mahony S, Deamant C (2022). Brain death determination and communication: an innovative approach using simulation and standardized patients. J Pain Symptom Manage.

[23] Metaxa V, Anagnostou D, Vlachos S, Arulkumaran N, Besemmane S, van Dusseldorp I, Aslakson RA, Davidson JE, Gerritsen RT, Hartog C, Curtis JR (2021). Palliative care interventions in intensive care unit patients. Intensive Care Med.

[24] Shelton W, Moore CD, Socaris S, Gao J, Dowling J (2010). The effect of a family support intervention on family satisfaction, length-of-stay, and cost of care in the intensive care unit. Crit Care Med.

[25] Curtis JR, Treece PD, Nielsen EL, Gold J, Ciechanowski PS, Shannon SE, Khandelwal N, Young JP, Engelberg RA (2016). Randomized trial of communication facilitators to reduce family distress and intensity of end-of-life care. Am J Respir Crit Care Med.

[26] Burns JP, Mello MM, Studdert DM, Puopolo AL, Truog RD, Brennan TA (2003). Results of a clinical trial on care improvement for the critically ill. Crit Care Med.

[27] Lamba S, Murphy P, McVicker S, Harris Smith J, Mosenthal AC (2012). Changing end-of-life care practice for liver transplant service patients: structured palliative care intervention in the surgical intensive care unit. J Pain Symptom Manage.

[28] Aslakson RA, Reinke LF, Cox C, Kross EK, Benzo RP, Curtis JR (2017). Developing a research agenda for integrating palliative care into critical care and pulmonary practice to improve patient and family outcomes. J Palliat Med.

[29] Daly K, Kleinpell RM, Lawinger S, Casey G (1994). The effect of two nursing interventions on families of ICU patients. Clin Nurs Res.

[30] Lautrette A, Darmon M, Megarbane B, Joly LM, Chevret S, Adrie C, Barnoud D, Bleichner G, Bruel C, Choukroun G, Curtis JR, Fieux F, Galliot R, Garrouste-Orgeas M, Georges H, Goldgran-Toledano D, Jourdain M, Loubert G, Reignier J, Saidi F, Souweine B, Vincent F, Barnes NK, Pochard F, Schlemmer B, Azoulay E (2007). A communication strategy and brochure for relatives of patients dying in the ICU. N Engl J Med.

[31] Jacobowski NL, Girard TD, Mulder JA, Ely EW (2010). Communication in critical care: family rounds in the intensive care unit. Am J Crit Care.

[32] White DB, Buddadhumaruk P, Arnold RM (2018). Family-support intervention in the ICU. N Engl J Med.

[33] Lilly CM, De Meo DL, Sonna LA, Haley KJ, Massaro AF, Wallace RF, Cody S (2000). An intensive communication intervention for the critically ill. Am J Med.

[34] Lilly CM, Sonna LA, Haley KJ, Massaro AF (2003). Intensive communication: four-year follow-up from a clinical practice study. Crit Care Med.

[35] Hatler CW, Grove C, Strickland S, Barron S, White BD (2012). The effect of completing a surrogacy information and decision-making tool upon admission to an intensive care unit on length of stay and charges. J Clin Ethics.

[36] Quenot JP, Rigaud JP, Prin S, Barbar S, Pavon A, Hamet M, Jacquiot N, Blettery B, Herve C, Charles PE, Moutel G (2012). Impact of an intensive communication strategy on end-of-life practices in the intensive care unit. Intensive Care Med.

[37] Carson SS, Cox CE, Wallenstein S, Hanson LC, Danis M, Tulsky JA, Chai E, Nelson JE (2016). Effect of palliative care-led meetings for families of patients with chronic critical illness: a randomized clinical trial. JAMA.

[38] Holloran SD, Starkey GW, Burke PA, Steele G, Forse RA (1995). An educational intervention in the surgical intensive care unit to improve ethical decisions. Surgery.

[39] Saft HL, Richman PS, Berman AR, Mularski RA, Kvale PA, Ray DE, Selecky P, Ford DW, Asch SM (2014). Impact of critical care medicine training programs' palliative care education and bedside tools on ICU use at the end of life. J Grad Med Educ.

[40] Rich C TT, Marsh J. Positive impact of palliative care education on ICU nurses: Center for Advanced Palliative Care; 2020 [cited 2021 November 20]. Available from: https://www.capc.org/blog/positive-impact-palliative-care-education-icu-nurses/.

[41] Oppenheim IM, Lee EM, Vasher ST, Zaeh SE, Hart JL, Turnbull AE (2020). Effect of intensivist communication in a simulated setting on interpretation of prognosis among family members of patients at high risk of intensive care unit admission: a randomized trial. JAMA Netw Open.

[42] Davidson JE, Aslakson RA, Long AC, Puntillo KA, Kross EK, Hart J, Cox CE, Wunsch H, Wickline MA, Nunnally ME, Netzer G, Kentish-Barnes N, Sprung CL, Hartog CS, Coombs M, Gerritsen RT, Hopkins RO, Franck LS, Skrobik Y, Kon AA, Scruth EA, Harvey MA, Lewis-Newby M, White DB, Swoboda SM, Cooke CR, Levy MM, Azoulay E, Curtis JR (2017). Guidelines for family-centered care in the neonatal, pediatric, and adult ICU. Crit Care Med.

[43] Vital Talk: Vital Talk; 2018 [cited 2018 09/27]. Available from: http://vitaltalk.org/.

[44] Arnold RM, Back AL, Barnato AE, Prendergast TJ, Emlet LL, Karpov I, White PH, Nelson JE (2015). The critical care communication project: improving fellows' communication skills. J Crit Care.

[45] Grudzen CR, Emlet LL, Kuntz J, Shreves A, Zimny E, Gang M, Schaulis M, Schmidt S, Isaacs E, Arnold R (2016). EM Talk: communication skills training for emergency medicine patients with serious illness. BMJ Support Palliat Care.

[46] Onishi E, Nakagawa S, Uemura T, Shiozawa Y, Yuasa M, Ito K, Kobayashi Y, Ishikawa H, Ouchi K (2021). Physicians’ perceptions and suggestions for the adaptation of a US-based serious illness communication training in a non-US culture: a qualitative study. J Pain Symptom Manage.

[47] Ito K, Uemura T, Yuasa M, Onishi E, Shiozawa Y, Ishikawa H, Ouchi K, Nakagawa S. The feasibility of virtual vitaltalk workshops in Japanese: can faculty members in the US effectively teach communication skills virtually to learners in Japan? Am J Hosp Palliat Care. 2021:10499091211044477. 10.1177/10499091211044477. 10.1177/10499091211044477 PMC8901778 34493061

[48] Curtis JR, Treece PD, Nielsen EL, Downey L, Shannon SE, Braungardt T, Owens D, Steinberg KP, Engelberg RA (2008). Integrating palliative and critical care: evaluation of a quality-improvement intervention. Am J Respir Crit Care Med.

[49] Curtis JR, Nielsen EL, Treece PD, Downey L, Dotolo D, Shannon SE, Back AL, Rubenfeld GD, Engelberg RA (2011). Effect of a quality-improvement intervention on end-of-life care in the intensive care unit: a randomized trial. Am J Respir Crit Care Med.

[50] Weiner SB (2020). Creating and implementing a resident emotional wellness initiative in an acute care setting: the role of the palliative care social worker. J Soc Work End Life Palliat Care.

[51] Hough CL, Hudson LD, Salud A, Lahey T, Curtis JR (2005). Death rounds: end-of-life discussions among medical residents in the intensive care unit. J Crit Care.

[52] Smith L, Hough CL (2011). Using death rounds to improve end-of-life education for internal medicine residents. J Palliat Med.

[53] Halpern SD (2018). Using default options and other nudges to improve critical care. Crit Care Med.

[54] Halpern SD, Becker D, Curtis JR, Fowler R, Hyzy R, Kaplan LJ, Rawat N, Sessler CN, Wunsch H, Kahn JM, American Thoracic S, American Association of Critical-Care N, Society of Critical Care M (2014). An official American Thoracic Society/American Association of Critical-Care Nurses/American College of Chest Physicians/Society of Critical Care Medicine policy statement: the Choosing Wisely(R) Top 5 list in Critical Care Medicine. Am J Respir Crit Care Med.

[55] Gershengorn HB, Garland A, Kramer A, Scales DC, Rubenfeld G, Wunsch H (2014). Variation of arterial and central venous catheter use in United States intensive care units. Anesthesiology.

[56] Cornia PB, Amory JK, Fraser S, Saint S, Lipsky BA (2003). Computer-based order entry decreases duration of indwelling urinary catheterization in hospitalized patients. Am J Med.

[57] Campbell ML, Guzman JA (2004). A proactive approach to improve end-of-life care in a medical intensive care unit for patients with terminal dementia. Crit Care Med.

[58] Schneiderman LJ, Gilmer T, Teetzel HD, Dugan DO, Blustein J, Cranford R, Briggs KB, Komatsu GI, Goodman-Crews P, Cohn F, Young EW (2003). Effect of ethics consultations on nonbeneficial life-sustaining treatments in the intensive care setting: a randomized controlled trial. JAMA.

[59] Turnbull AE, Krall JR, Ruhl AP, Curtis JR, Halpern SD, Lau BM, Needham DM (2014). A scenario-based, randomized trial of patient values and functional prognosis on intensivist intent to discuss withdrawing life support. Crit Care Med.

[60] Treece PD, Engelberg RA, Crowley L, Chan JD, Rubenfeld GD, Steinberg KP, Curtis JR (2004). Evaluation of a standardized order form for the withdrawal of life support in the intensive care unit. Crit Care Med.

[61] Hall RI, Rocker GM, Murray D (2004). Simple changes can improve conduct of end-of-life care in the intensive care unit. Can J Anaesth.

[62] Norton SA, Hogan LA, Holloway RG, Temkin-Greener H, Buckley MJ, Quill TE (2007). Proactive palliative care in the medical intensive care unit: effects on length of stay for selected high-risk patients. Crit Care Med.

[63] Carlson RW, Devich L, Frank RR (1988). Development of a comprehensive supportive care team for the hopelessly ill on a university hospital medical service. JAMA.

[64] White DB, Angus DC, Shields AM, Buddadhumaruk P, Pidro C, Paner C, Chaitin E, Chang CH, Pike F, Weissfeld L, Kahn JM, Darby JM, Kowinsky A, Martin S, Arnold RM, Investigators P (2018). A randomized trial of a family-support intervention in intensive care units. N Engl J Med.

[65] Hebert RS, Prigerson HG, Schulz R, Arnold RM (2006). Preparing caregivers for the death of a loved one: a theoretical framework and suggestions for future research. J Palliat Med.

[66] Wendler D, Rid A (2011). Systematic review: the effect on surrogates of making treatment decisions for others. Ann Intern Med.

[67] Kirchhoff KT, Beckstrand RL (2000). Critical care nurses’ perceptions of obstacles and helpful behaviors in providing end-of-life care to dying patients. Am J Crit Care.

[68] Nelson JE (2006). Identifying and overcoming the barriers to high-quality palliative care in the intensive care unit. Crit Care Med.

[69] Fassier T, Lautrette A, Ciroldi M, Azoulay E (2005). Care at the end of life in critically ill patients: the European perspective. Curr Opin Crit Care.

[70] Dietz I, Plog A, Jox RJ, Schulz C (2014). “Please describe from your point of view a typical case of an error in palliative care”: Qualitative data from an exploratory cross-sectional survey study among palliative care professionals. J Palliat Med.

[71] Dietz I, Borasio GD, Molnar C, Muller-Busch C, Plog A, Schneider G, Jox RJ (2013). Errors in palliative care: kinds, causes, and consequences: a pilot survey of experiences and attitudes of palliative care professionals. J Palliat Med.

[72] Gallagher J, Byrne S, Woods N, Lynch D, McCarthy S (2014). Cost-outcome description of clinical pharmacist interventions in a university teaching hospital. BMC Health Serv Res.

